# Clusters of Ancestrally Related Genes That Show Paralogy in Whole or in Part Are a Major Feature of the Genomes of Humans and Other Species

**DOI:** 10.1371/journal.pone.0035274

**Published:** 2012-04-26

**Authors:** Michael B. Walker, Benjamin L. King, Kenneth Paigen

**Affiliations:** 1 The Jackson Laboratory, Bar Harbor, Maine, United States of America; 2 Mount Desert Island Biological Laboratory, Salisbury Cove, Maine, United States of America; Hospital for Sick Children, Canada

## Abstract

Arrangements of genes along chromosomes are a product of evolutionary processes, and we can expect that preferable arrangements will prevail over the span of evolutionary time, often being reflected in the non-random clustering of structurally and/or functionally related genes. Such non-random arrangements can arise by two distinct evolutionary processes: duplications of DNA sequences that give rise to clusters of genes sharing both sequence similarity and common sequence features and the migration together of genes related by function, but not by common descent [1], [2], [3]. To provide a background for distinguishing between the two, which is important for future efforts to unravel the evolutionary processes involved, we here provide a description of the extent to which ancestrally related genes are found in proximity.

Towards this purpose, we combined information from five genomic datasets, InterPro, SCOP, PANTHER, Ensembl protein families, and Ensembl gene paralogs. The results are provided in publicly available datasets (http://cgd.jax.org/datasets/clustering/paraclustering.shtml) describing the extent to which ancestrally related genes are in proximity beyond what is expected by chance (i.e. form paraclusters) in the human and nine other vertebrate genomes, as well as the *D. melanogaster*, *C. elegans*, *A. thaliana*, and *S. cerevisiae* genomes.

With the exception of *Saccharomyces*, paraclusters are a common feature of the genomes we examined. In the human genome they are estimated to include at least 22% of all protein coding genes. Paraclusters are far more prevalent among some gene families than others, are highly species or clade specific and can evolve rapidly, sometimes in response to environmental cues. Altogether, they account for a large portion of the functional clustering previously reported in several genomes.

## Introduction

Contemporary arrangements of genes along chromosomes are the products of mutation, viewed as genomic changes at all levels, followed by natural selection, and we can expect that preferable arrangements are likely to prevail over the span of evolutionary time. In contrast to prokaryote genomes that are enriched with clusters of functionally related but structurally distinct genes organized in operons [4], a significant fraction of eukaryotic genomes consists of clusters of structurally and often functionally related genes that are related by sharing a common ancestral genomic duplication.

Several previous efforts to describe clustering of structurally related genes have employed an all-against-all protein-level sequence similarity analysis to estimate the genome wide metrics of such clusters in eukaryotic species, especially vertebrates. Some have focused on the description of tandemly arrayed genes (TAGs), reporting that an average of 14% of genes in vertebrates are clustered in that way [5], with similar metrics found for A. thaliana [6], but only 2% so clustered in yeast [7]. The outcome of these approaches depends on both the choice of similarity searching program (e.g., BLASTP) parameters regarding sensitivity and the degree to which like sequences are clustered. Bretaudeau, Sallou,and Lecerf increased the sensitivity of their analysis using modified BLASTP parameters in order to detect domain level sequence similarities and expanded the scope of gene clusters to include all structurally related genes residing within 2.5 MB of each other. They reported that among the vertebrates tested, an average of 30% of genes are present in structural clusters (http://dgd.geneoust.org) [8]. Unfortunately, this study did not test for the statistical significance of finding that genes sharing a domain are located in proximity; and it is unclear how much of the clustering detected was due to chance alone. As we show below, this is a particularly acute problem for high frequency domains that are likely to occur near each other by chance, and very likely led to an overestimate of structural clustering. Conversely, in all such studies it is unclear how much information regarding gene clustering is missed due to the high levels of sequence divergence that arises between those ancestrally duplicated genes that have remained in proximity since the common ancestor of all vertebrates and even longer; detecting such arrangements requires the use of more sensitive approaches such as those involving Hidden Markov Models. In this present study our aim is to obtain new metrics and catalogues of clustered genes that both share structural features detected at the domain level and are highly significant in their proximity to one another, while allowing for sequence divergence to extend to the superfamily level.

Several other previous studies have examined clusters of functionally related genes defined by their shared expression patterns, functional pathway enrichment or shared Gene Ontology (GO) annotations [9], [10], [11], [12]. These efforts attempted to distinguish whether such clusters arose by proximate gene duplications or by migration together at a common site by correcting for the presence of tandemly duplicated genes recognized by their sequence similarity. However, as we show below, a comprehensive description of genomic functional organization also requires considering clusters of ancestrally related genes that are difficult to recognize by sequence similarity methods alone or because they include significant numbers of interspaced, unrelated (interstitial) genes.

Because duplications of genomic material provided a key evolutionary step in the origin of chromosomal arrangements, in this analysis we have applied the concept of paralogy, shared ancestry, to include genes that share a duplicated region recognizable as a common functional domain that arose as an domain duplication, as well as including genes that obviously share their entire sequence as a consequence of a prior, full gene duplication. Recent advancements in both the quality and quantity of gene annotation data have made it possible to assert gene paralogy by combining data from five sources of structural annotation data. We use the term paraclusters to represent clusters of paralogous genes that share sufficient structural similarity to imply a common ancestry, either in their entirety or in the possession of a common functional domain, and that are located in close proximity, where proximity is defined by a low probability that they would occur within the same neighborhood by chance. The long-term maintenance of these arrangements depends on the balance between possible purifying selection to maintain a functional advantage and random genomic rearrangements that promote disruption and dispersion. Possible selective pressures promoting adjacency include retention within clusters sharing common regulatory elements and/or retention as a means of promoting the co-inheritance of co-adapted sets of alleles among these genes.

Gene duplications offer evolutionary opportunities for creation and/or specialization of gene function. One of the duplicates is now free to acquire new molecular specificity by altering its binding properties, change its response to regulatory signals that can alter tissue specificity of expression, mutate its transcript splicing patterns to produce distinctly new molecular forms of protein, or even divide its molecular functions between the two duplicates by having each partner lose complementary functional domains [13]. Duplicated genes within clusters also provide a unique environment for genomic phenomena that may not be found among dispersed duplicates. In addition to the possibility of shared regulatory elements, there is the potential co-inheritance of allelic combinations, which in the extreme case can lead to near total linkage disequilibrium of alleles within clusters, the expansion and contraction of tandem gene copy number, and gene conversion events which can act to either homogenize or diversify the DNA sequences of clustered genes.

Whatever the order and types of genomic events that have occurred, and whatever evolutionary forces have been operative, it is clear that together they have left contemporary genomes with extensive genomic clustering of paralogous genes. In the human genome, for example, we find that greater than one in five genes are organized in this way, making clustering an important feature of contemporary genomic organization and providing an impetus to understand the features of these arrangements, how they might affect genomic function, and what role if any they play in the survival of species [14]. As a means of describing some of the general characteristics of paraclusters and providing an evolutionary perspective, we have integrated the information from multiple datasets to create catalogs of the paraclusters present in a diverse set of vertebrates (*Homo sapiens, Macaca mulatta, Pan troglodytes, Mus musculus, Rattus norvegicus, Canis familiaris, Bos taurus, Monodelphis domestica, Gallus gallus* and *Danio rerio*), two invertebrates, *Drosophila melanogaster* and *Caenorhabditis elegans*, the higher plant *Arabidopsis thaliana* and the yeast *Saccharomyces cerevisiae*. Overall genome statistics for each of the species tested are listed in Table S1.

## Results

### Defining paralogs and paraclusters

Our analysis of these 14 genomes relies upon a robust classification of paralogous genes and we used a total of five datasets for this purpose. Two of these datasets primarily define protein relatedness by global protein sequence similarity, and the other three by shared functional domains. The Ensembl families dataset employs an all-against-all protein sequence similarity search on all Ensembl protein predictions of all represented species using BLASTP and classifies those results using Markov Chain Clustering [15]. The PANTHER superfamily resource classifies genes and proteins by their functions utilizing Hidden Markov Models and manual curation [16]. For these datasets, we have assumed paralogy when two genes in proximity are assigned to the same protein family. The third structural dataset is the Ensembl paralogs dataset which is derived using the TreeBeST algorithm [17]; there, paralogy is asserted explicitly.

Two additional datasets place their emphasis on the presence of shared functional domains, relying on Hidden Markov Models for representing structural features. Here we have imputed paralogy when two proteins share domains and are located in close proximity beyond chance expectation, which depends on the frequency of the domains across the entire genome. The SCOP superfamilies dataset uses domain classification to assert common evolutionary origin between proteins even with low sequence similarity [18], [19], [20]. The InterPro dataset integrates many classification systems of protein signatures or domain structures into a single source [21].

Operationally, we define a paracluster as a group of *p* paralogous genes (defined using one of the five datasets) that occur together within a span of *n* genes with a less than 0.01 expectation of achieving that level of clustering by chance anywhere across the entire genome. Probabilities are calculated using the hypergeometric distribution, which estimates the chance probability of seeing *p* paralogous genes within a span of *n* successive genes along a chromosome given the total number of genes sharing a specific annotation and the total number of genes in the genome (see Methods section). We corrected for the number of opportunities for seeing such a cluster, which for all practical purposes equals the number of genes in a genome. An expectation value of e<0.01 (p-value<0.01/n, where n∼ = total gene count) was used to reduce false positives. This approach tends to underestimate the number of paraclusters detected, making our estimates of the extent of paraclustering relatively conservative. A considerable majority of the paraclusters we found derived from whole gene duplications, consisting of members that are paralogous according to the Ensembl paralogs dataset. However there is an additional group, somewhat less than one-sixth the total, the exact value depending on the species, that derived from local duplications of functional domains or whole gene duplications that have highly diversified in sequence. To assert orthology of paraclusters between species we used data obtained from the InParanoid database which distinguishes in-paralogs (gene duplications that arose after speciation) from out-paralogs (those that arose before speciation) [22].

### Paracluster dimensions

**Figure 1:**
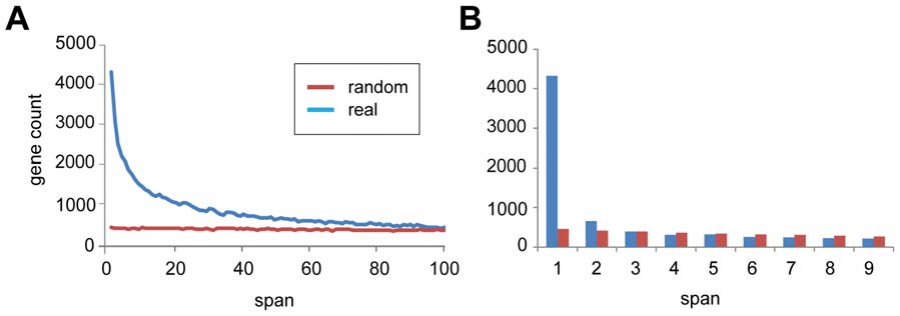
Frequency distribution of distances between structurally related genes. (a) Frequencies of distances between structurally related genes across the human genome (blue) as compared to the same data generated from randomly permuted genomes (red). (b) Frequencies of distances to the nearest structurally related gene for all human genes (blue) as compared to the same data generated from randomly permuted genomes (red).

To provide a first level, genome wide description of proximity among genes sharing structural features, a master list of protein coding genes for each genome was assembled with the genes placed in rank order by their locations along chromosomes beginning with the first gene on chromosome 1 and proceeding to the end of the last gene on the Y or the smallest chromosome as the case may be. Describing intergenic distances by differences in rank order rather than base pairs of DNA sequence served both to avoid statistical artifacts arising from variations in gene density along chromosomes and to preserve the essential feature of relative positioning along chromosomes. Proximity metrics of structurally related genes were tabulated for each dataset by taking each gene in turn and asking whether the gene *n* genes further away along the chromosome is structurally related. The resulting distributions for the human genome, compared with the average of ten control analyses using gene lists randomly permuted for gene order, are presented in Figures 1 A and B, which describe whether the gene *n* genes away is structurally related, and the distance to the closest structurally related gene. A few very large families of genes have a disproportionate impact on these results. Removing only two very large clustered families from the analysis, the zinc finger C2H2 genes on chromosome 19 and the G protein receptor genes (GPCR) genes on chromosome 11, strongly reduced the likelihood of finding a gene with structural similaries at more distant locations. The 263 C2H2 genes on human chromosome 19 in our analysis were present in 11 clusters, all of them consisting of quasi-tandem arrays of genes with diverse sequence similarities between them and containing a few small gaps 1 to 3 genes long, with only a few gaps of up to 8 genes. A few of these larger gaps actually contained nested tandem arrays of different families. The 191 G-protein coupled receptors on chromosome 11 were present in 5 clusters, three on the p arm and two on the q arm. These clusters had similar arrangements as the C2H2 gene clusters, with few gaps that were either very small in size or somewhat larger due to nested clusters of genes from different families. Also similar to the C2H2 gene clusters, the G-protein coupled receptor clusters contained many genes with sufficiently diverse sequences such that their paralogy annotations reflect gene homologies at the subfamily level of gene classification.

**Figure 2:**
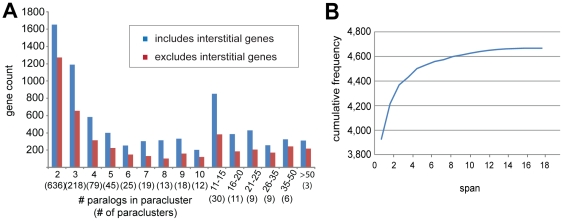
Paracluster sizes. (a) Total gene count in paraclusters as a function of paracluster size measured as the number of paralogs in each paracluster. Gene counts include (blue) and exclude (red) interstitial genes. The parenthetical text provides the number of paraclusters having the corresponding size. (b) Cumulative frequency distribution of distances (spans) between genes sharing the same paracluster.

Table 1 provides a matrix describing for the human genome the numbers of paracluster genes found in common among the various datasets. Table S2 provides that information for all species. Figure 2A presents the total number of genes (both including and excluding interstitial genes) in human paraclusters of various sizes measured by the number of paralogs they contain. Figure 2B describes the cumulative number of genes present in human paraclusters of various sizes, reaching a level of 22.4% of the genome.

**Table 1:** Counts of human paracluster genes found in common between datasets.

H. sapiens	Ensembl paralogs	Ensembl family	PANTHER	SCOP	InterPro	
4,638 genes	3,861	2,041	2,052	1,806	2,712	Ensembl paralogs
		2,195	1,018	1,138	1,599	Ensembl family
			2,311	1,319	1,788	PANTHER
				2,227	2,063	SCOP
					3,269	InterPro

The summary for the human and mouse genomes of the numbers of paraclusters and the numbers of genes they contain using each dataset and the combination of all datasets are provided in Table 2. Inspection of the data indicates that the InterPro and SCOP datasets tend to merge adjacent clusters that are seen as separate by the two Ensembl datasets and PANTHER, so that the different datasets find similar total numbers of genes present in paraclusters, albeit with different average paracluster sizes.

**Table 2:** Counts of human/mice paracluster genes found from each dataset.

species	database	paracluster count	genes in paraclusters	percent genes in paraclusters
*H. sapiens*	Ensembl paralogs	1,162	3,861	18.7
	Ensembl family	648	2,195	10.6
	PANTHER	629	2,311	11.2
	SCOP	345	2,227	10.8
	InterPro	660	3,269	15.8
	merged	1,133	4,638	22.4
*M. musculus*	Ensembl paralogs	1,149	5,074	22.3
	Ensembl family	755	3,689	16.2
	PANTHER	713	2,951	16.0
	SCOP	391	3,696	16.2
	InterPro	646	4,497	19.7
	merged	1,100	5,887	25.8

It is apparent that paralog clustering is a major feature of the human genome. The combined human data indicates that among the 22.4% of all human genes in paraclusters, almost all were either directly adjacent (20.1%) or within 10 genes of another family member (22.1%). Using the Ensembl paralogs dataset, which permits the calculation, we find that among the subset of human genes that have at least one other paralog present in the genome, and thus could potentially participate in forming a paracluster, more than a quarter (26.05%) do so. In this connection, we should emphasize that, as we show below, the likelihood of finding a paralog nearby differs dramatically for different gene families, a point that is of special importance for genome wide association studies attempting to map the location of quantitative trait loci important in human health and disease.

### Differences between data sets

Although all five data sources are based on structural information, there are significant differences among them in their ability to detect paraclusters. The Ensembl families and paralogs datasets are more stringent with respect to the level of overall sequence similarity required to identify paralogs, which limits their ability to detect clustering among highly divergent sequences that have nevertheless remained in proximity over evolutionary time. For example, Ensembl build 58 of the human genome sequence includes 20,665 protein coding genes distributed among 16,241 Ensembl gene families, with a small number of genes having alternate transcripts assigned to more than one family. Among them, small families predominate; 19,846 genes (96%) are in families with 10 members or less; 14,116 belong to families with only a single member. Among the families with multiple members, there are 6,549 genes distributed among 2,125 families (ave. 3.1 per family), with only 877 genes (4%) belonging to families with greater than 10 members. The same is true of the PANTHER dataset. However, many ancient paraclusters that were present in the common ancestors of all the vertebrate species we tested are captured using the SCOP superfamily and InterPro domains datasets. An instructive example is provided by the immunoglobulin superfamily of genes whose members have highly diverged from one another in both sequence and function; their clustering at both local and broad scales is readily detected using the SCOP and InterPro datasets, which emphasized domain sharing, but is not detected in its entirety using the Ensembl datasets or even the PANTHER super family datasets.

In contrast, the sequence based Ensembl datasets detected paraclusters that the InterPro/SCOP datasets failed to recognize as significant. The major reason for this latter failure is the statistical constraint imposed by the enormous numbers of copies of certain domains, such as those present in G protein coupled receptors and immunoglobulin proteins, which increase the level of proximity required to meet the threshold of *e*<0.01, resulting in a higher proportion of false negatives from the domain datasets. A secondary reason for failure is the existence of uncharacterized genes that have been identified in the assembly build but as yet have no known reference and lack annotation details, leaving them outside the scope of InterPro and SCOP; these can be recognized as paralogs by datasets based on sequence similarities alone, although some of these are eventually retracted or identified as non-transcribed pseudogenes in subsequent assembly builds.

The consequence of choosing one dataset over another is that even within the same species paraclusters can appear more or less extensive, or even go entirely undetected, depending on how they are annotated in one dataset or another. In part, we can compensate for these deficiencies by merging the results obtained using the multiple datasets; this allows individual paraclusters to be more fully realized in that separate portions of a paracluster may be recognized by different datasets, with the complete paracluster only revealed by merging the results. The principal reasons for this last effect are the lack of gene annotation in one or another dataset; the limitations inherent in setting statistical thresholds for the automated sequence similarity approaches applied by the Ensembl pipelines, and finally, the differential impact among datasets that gene family size differences have on probability calculations when applying the hypergeometric distribution. Given that all five sources of information have their limitations and that we have taken a stringent approach to measuring statistical significance, we have considered genes as existing in paraclusters when any of the five datasets reported so. For these reasons our software was developed so that it can be easily extended to include other systems of structural annotation. We examined those genes which were exclusively added by any one system and found their nomenclature to be predominately that of the rest of the paracluster suggesting that the merging overcomes missing annotations and false negative detection within any one system. This was particularly true of genes that were exclusively defined through the Panther, and the Ensembl protein families and paralogies datasets.

Despite the differences between datasets, as shown in Tables 1 and 2, there is a great deal of overlap among genes that are assigned to paraclusters using the different datasets. Indeed, the Ensembl paralogs dataset detects as clustered paralogs the great majority of genes detected as such by any of the datasets (18.7%/22.4% in humans).

### The impact of including domains-only databases

**Figure 3:**
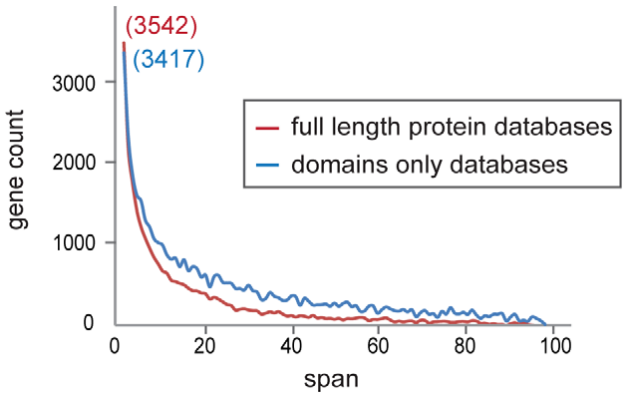
Metrics based on whole gene sequence similarity versus common domains. Frequencies of distances between genes within paraclusters as described by the combination of data from PANTHER, Ensembl family and Ensembl paralogy datasets (red) and as described by the combination of a data from SCOP and InterPro datasets (blue). The numbers in parentheses are the number of paraclusters with only two genes.

To better understand the nature of the differences between assessing paralogy arising from whole gene duplications and that arising from domain shuffling or involving genes whose ancestry is only evidenced at the superfamily level, we contrasted the paraclusters found exclusively using the PANTHER, Ensembl families and Ensembl paralog datasets, that emphasize full length protein sequence to infer homology, with those found exclusively using SCOP and InterPro, that rely on conserved protein domains (Figure 3). These represented a total of 52 paraclusters. Of these, 10 were determined to be due to annotation errors in build 58 which were subsequently corrected in the current build, or represented genes that were retracted subsequent to build 58. Among the 42 remaining paraclusters, 8 were actually annotated as paralogous by the Ensembl database, but did not meet the stringent e<0.01 expectation cutoff, but did meet a criterion of e<0.05. An additional paracluster of two tandem genes was annotated as belonging to the same PANTHER superfamily, but only reached an expectation threshold of e<0.15. Filtering out these cases, left 33 paraclusters defined exclusively by InterPro and/or SCOP (Table S3).

In order to better understand their origins, we classified each cluster in terms of its superfamily domain organization and determined the last common ancestors when possible by evaluating the synteny across species utilizing Ensembl ortholog data. We also checked to see if each cluster contained more than one member with an ortholog or paralog whose origin appeared to predate the oldest common ancestor of the cluster, reasoning that migratory clustering was less likely if the required orthologs or paralogs did not exist prior to the origin of the paracluster. Unfortunately, in some cases the oldest common ancestor possessing the cluster could not be found due to incomplete assembly mapping in low coverage genomes. Table S3 presents the results of these tests suggesting that many within this group of paraclusters show evidence for arising by local duplication. Most of these clusters contained genes whose superfamily domain architecture matched across the entire sequence. Other patterns were found that suggest gene fusion, whereby a gene shares half its domain architecture with one neighbor and half with another with no similarities existing between the two neighbors, and domain shuffling, whereby a gene acquires a domain from a nearby cluster indicated by the observation that no paralogs of the particular gene located elsewhere in the genome possess the acquired domain. Only a couple of cases involved genes with mixed domains whereby only one domain is shared with the other cluster members and the rest of the domains are different and unrelated. One prevalent characteristic amongst most of these clusters is that their genes share low sequence similarity, even within those clusters where the superfamily domains match across the entire protein. Some of these clusters have gene pairs for which no significant sequence similarity can be found. These clusters were found to involve genes belonging to families that are conserved only at the level of the protein fold and have little sequence conservation across the entire protein [23], [24], [25], [26]. One interesting aspect of this list of paraclusters is that more than half of them have ancestral relationships with other paraclusters mostly coinciding within the list, suggesting that the evolution of these genes involve processes other than simple tandem duplication and may represent the outcomes of ancient regions of duplicated genes becoming dispersed across the genome into sub clusters or possible duplication of regions involving whole clusters or even whole genome duplications.

Just as we looked for whole clusters which were exclusively detected using the domains dataset, we also looked for single genes likewise exclusively detected which extended the range of some paraclusters. Evaluating these genes uncovered 11 erroneous genes having annotation errors from build 58 that were subsequently corrected and around 30 genes were included within large paraclusters which only shared a single domain with the other paracluster members but otherwise had completely different structures and appeared unrelated. We attributed this to a “gravitational effect” whereby the cluster was large enough and the matching domain was prolific enough to make nearby genes look significant in their proximity. A few cases of domain shuffling and gene fusion were found, but the vast majority of these genes shared a domain organization with the other members across their entire length. Most genes have nomenclatures properly corresponding to the clusters yet have sequences which have highly diverged, resulting in many clusters having genes with consist nomenclature yet whose annotations of paralogy reflect homology at the subfamily level only. Examples of paraclusters of this type include olfactory receptors, C2H2 zinc finger genes, immune system genes, protocadherins, histones, HOX clusters, cytochrome P450s, keratins, neurotransmitter receptors, kallekreins, serine peptidase inhibitors and cytokines. Most of these classes involve clustered genes having undergone lineage-specific gains and losses, a contributing factor to the diversification of gene families.

### Species similarities in paracluster content

Table 3 summarizes the numbers of paraclusters and their gene content for all of the species tested. Table S4 provides the breakdown of results for each dataset in each species. With some variation, the basic picture seen in humans extends across all of the species examined, both animal and plant, with the sole exception of *S. cerevisiae*. Multicellular organisms show similar fractions of their genes in paraclusters and similar dimensions of paraclusters.

**Table 3:** Counts of paracluster genes from each species.

species	total genes	paracluster count	genes in paraclusters	percent genes in paraclusters
*H. sapiens*	20,686	1,133	4,638	22.4
*P. troglodytes*	19,199	925	3,622	18.9
*M. mulatta*	21,023	1,125	4,090	19.5
*M. musculus*	22,793	1,100	5,887	25.8
*R. norvegicus*	22,925	1,160	5,930	25.9
*C. familiaris*	19,014	1,111	4,070	21.4
*B. taurus*	19,030	1,120	4,450	23.4
*M. domestica*	18,640	970	4,161	22.3
*G. gallus*	15,310	669	1,962	12.8
*D. rerio*	22,940	1,734	5,820	25.4
*D. melanogaster*	13,858	976	3,088	22.3
*S. cerevisiae*	6,666	85	217	3.3
*C. elegans*	20,212	1,423	5,213	25.8
*A. thaliana*	31,070	1,569	5,000	16.1

**Figure 4:**
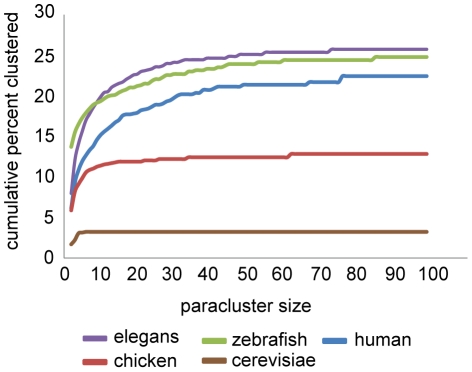
Species comparison of genome wide clustering metrics. Cumulative frequency distribution of the percentage of genes in paraclusters within the genomes of a selected set of species as a function of paracluster sizes.

The chicken genome is the notable exception among vertebrates. The lower percentage of chicken genes found in paraclusters is not the consequence of incomplete annotation as only 8% of genes lack annotation in that species. Nor is it due to the presence of a large number of very small chicken chromosomes, which act to divide the genome up into small fragments. Indeed, four of the largest chicken paraclusters were found on micro chromosomes; two keratin clusters, and an immunoglobulin cluster on chromosome 27 and the Major Histocompatibility Complex on chromosome 16. And on a genome wide scale, there was no difference in the density of paraclusters (numbers of paraclusters per Mb of DNA) in small v. large chromosomes, or indeed among all chromosomes (data not shown). Rather, the relative lack of paraclusters in chickens reflects a paucity of the common, large gene family expansions that characterize mammals (Figure 4). Even homologous clusters that were relatively large in the chicken showed even greater expansions in human and other mammalian species; for example, an olfactory receptor cluster containing only 8 genes in the chicken is homologous to a cluster with 74 genes in human and 161 in mouse. This lack of larger paraclusters is likely related to the substantial reduction in segmental duplications and pseudogenes found in general in the chicken genome [27]. Whether or not this is a common feature of avian species or limited to a narrower clade can only be determined when annotated genomes of other avian species become available,

### Species differences in paracluster identity

Species comparisons suggest considerable birth and death of paracluster arrangements over evolutionary time and the importance of species specific expansions of gene families [28], [29] (Table 4). Although more than 75% of human paraclusters were present in the common ancestors of primates (Table 5), only somewhat more than half of human paraclusters are commonly present in both chimpanzees and macaques, suggesting considerable loss of paraclusters. Chimpanzees do presents a special case among the primates; the number of species specific genes in paraclusters (4.7%) being much lower than found in either macaque (17.2%) or human (18.0%). This is unlikely to be biologically correct; it more likely reflects the current state of the genome assembly for chimpanzee and the difficulty of detecting recent, species specific gene duplications that are quite similar in sequence and hence difficult to resolve using the current whole-genome shotgun approach [30].

**Table 4:** Percent of species specific paraclusters and common paraclusters within clades.

species	cluster count	species specific	common in all primates tested	common in all mammals tested	common in all vertebrates tested	common in all animals tested	common in all pecies tested
*H. sapiens*	1,133	18%	59%	31.7%	10.5%	0.6%	0.2%
*P. troglodytes*	925	4.7%	72.9%	39.1%	12.4%	0.7%	0.2%
*M. mulatta*	1,125	17.2%	59.4%	32.2%	10.1%	0.6%	0.2%
*M. musculus*	1,100	10%		33.6%	10.9%	0.9%	0.2%
*R. norvegicus*	1,160	14.1%		31.3%	9.7%	0.5%	0.2%
*C. familiaris*	1,111	17%		32.7%	10.4%	0.8%	0.2%
*B. taurus*	1,120	20.6%		31.6%	10.4%	0.8%	0.2%
*M. domestica*	970	14.1%		37.8%	12.3%	0.7%	0.2%
*G. gallus*	669	19.9%			16.1%	0.8%	0.2%
*D. rerio*	1,734	59.5%			6.9%	0.4%	0.2%
*D. melanogaster*	976	68%				1.1%	0.1%
*C. elegans*	1,423	78.5%				0.8%	0.1%
*S. cerevisiae*	85	74.1%					1.2%
*A. thaliana*	1,569	76%					0.1%

The ‘species specific’ column represents the percentage of paraclusters which have no in-paralogs clustered in any other species analyzed; the ‘common’ columns represent the percentage of paraclusters which have at least one in-paralog existing in a paracluster in all of the species analyzed within each clade.

**Table 5:** Percent of shared paraclusters within clades.

species	first detected in primates	first detected in mammals	first detected in vertebrates	first detected in animals
*H. sapiens*	23.7%	47.1%	79.1%	89.8%
*P. troglodytes*	10.9%	39.0%	76.2%	88.8%
*M. mulatta*	21.4%	45.7%	80.1%	91.5%
*M. musculus*		43.9%	75.5%	89.6%
*R. norvegicus*		46.2%	76.6%	91.2%
*C. familiaris*		44.7%	82.0%	92.3%
*B. taurus*		45.9%	78.1%	91.2%
*M. domestica*		34.2%	74.6%	90.0%
*G. gallus*			74.6%	88.3%
*D. rerio*			83.2%	93.5%
*D. melanogaster*				90.0%
*C. elegans*				93.0%

The percentage of paraclusters which have at least one in-paralog existing in a paracluster of any of the species analyzed within each clade.

On a broader scale, only 35% or so of paraclusters are common to all mammals tested, and only 12–13% of paraclusters are present in all vertebrates tested. The evolution of paraclusters is particularly apparent when the analysis is extended to the invertebrate *D. melanogaster* and *C. elegans* genomes, where only 0.8% of clusters are in common, and to *A. thaliana*, which lacks almost all contribution to paraclustering from animal specific protein domains such as those characterizing immunoglobulins or olfactory receptors.

### Effects of family size on paraclustering

**Figure 5:**
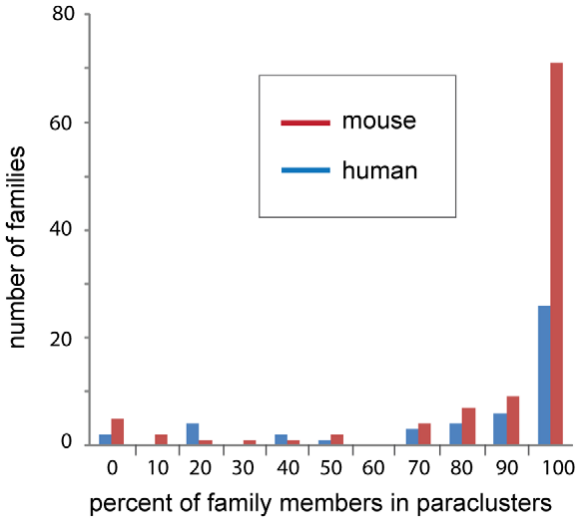
Extent of clustering within gene families. Frequencies of Ensembl gene family counts grouped by the percentage of family members contained within paraclusters (only families with 10 or more family members were counted).

Using the Ensembl families dataset, the effects of family size were pronounced in vertebrates, but not invertebrates and also not detectable in either using the SCOP dataset. In human, mouse and chicken there is a considerable association between Ensembl family size and the number of family members in paraclusters. In human, 75% of the 48 families with 10 or more members have over 80% of their genes in paraclusters, while only 33% of the 1998 families with five or less members show that much clustering (p<0.0005). This is almost certainly the result of recent family expansions in the vertebrates via tandem duplication events. This is particularly notable among olfactory receptors, zinc finger proteins, histones, keratins, PRAME family members, and vomeronasal receptors. Interestingly, the extent to which this has happened is also lineage dependent; mice have almost three times as many large families as humans [29] and nearly all are highly clustered (Figure 5).

### Evolutionarily Divergence

There is considerable diversity of paracluster identities among species; very few are widely represented. Among those that are, histone genes are unique in showing evidence of clustering in all of the species tested. Two histone paraclusters in humans contain multiple members of the four histone subfamilies required for nucleosome assembly: H3, H2B, H2A, and H4, while separate paraclusters exist containing the H1 histone genes which bind linker DNA between nucleosomes. Fruit flies possess a single cluster with over 100 tandemly arrayed genes with the various sub-families represented to very different extents. *A. thaliana* has two orthologous histone paraclusters, each containing only members of the H3 subfamily, while all other histone orthologs in *A. thaliana* remain unclustered. *S. cerevisiae* presents the simplest case, with only a single paracluster containing just one member from each subfamily except H1.

Only four classes of paraclusters other than histones were present in all multicellular species tested, suggesting they are under particular evolutionary constraints: glucuronosyltransferases, cytochrome P450s, members of the CAP superfamily and the homeodomain-like superfamily.. The glucuronosyltransferases and cytochrome P450 gene families are both involved in the metabolism of xenobiotics; the fact that those P450s involved in xenobiotic metabolism are clustered while those involved in intermediary metabolism are not, provides evidence that this is the evolutionary pressure maintaining clustering. In *A. thaliana* these families cluster prolifically; according to the SCOP superfamily datasets there are 77 P450 clusters present in *A. thaliana*. Orthologs were found in *S. cerevisiae*, however not in statistically significant clusters. In animals, HOX clusters containing members of the homeodomain-like superfamily are important regulators of embryonic development. In *A. thaliana* the homeodomain-like superfamily cluster contains a putative ancester of the HOX clusters having in-paralogs for the Evx1 and Evx2 genes, which are both significantly clustered with the HOXA and HOXD paraclusters respectively in vertebrates and whose linkage with HOX genes seems to represent the ancestral condition of the primordial metazoan Hox cluster [31], [32]. Finally, the CAP Superfamily consists of a diverse set of proteins of as yet unknown function, named according to the three domains that they contain, namely Cysteine-rich secretory proteins, Antigen 5, and Pathogenesis-related 1 [33].

The evolutionary origins of paraclusters can sometimes be deduced using the InParanoid database which describes gene to gene homology between any two represented species, distinguishing in-paralogs from out-paralogs. With respect to paraclusters, in-paralogs are those genes that have expanded within paraclusters since the two compared species diverged from a common ancestor, whereas out-paralogs already existed within the paraclusters of the common ancestor. Mapping the InParanoid datasets onto paraclusters it is apparent that there is almost complete turnover of paracluster gene members across different kingdoms and phylum, implicating many paracluster genes as species or clade specific. Even though 21% of human paraclusters contained at least one out-paralog in common with paraclusters of non-vertebrate species, only 1.6% of human clusters had two or more out-paralogs in common. This suggests that when paraclusters sharing a common ancestry are both undergoing expansion in two distantly related species, these expansions are largely independent processes in the two species. This agrees with our observation that vertebrate paraclusters are highly distinct in gene content from those found in the invertebrates, plants, and fungi. This is true even though some functional classes are conserved and some of the most prolific superfamilies are commonly present across all the species we tested. (Table S5).

### Clusters of paraclusters

Paraclusters sometimes exist in complex arrangements such as clusters of paraclusters, nested paraclusters and interlaced paraclusters, as though some chromosomal regions are especially prone to these types of arrangements. Many of these regions have undergone multiple rounds of local gene duplications that have remained in proximity while undergoing sequence divergence. Some regions appear held together by purifying selection, the genes within them operating together as a group, presumably under a coordinated regulatory mechanism. A prime example is the Epidermal Differentiation Complex, a 2 Mb paracluster of late cornified cell envelope genes on human Chr 1, [34] that consists of three paraclusters of mostly tandemly arrayed genes arranged such that two of the paraclusters are nested inside the third. The large outer surrounding cluster consists of 25 genes, all belonging to the same SCOP superfamily, EF-Hand. One of the inner paraclusters consists of only two tandem genes expressing peptidoglycan recognition proteins; while the other nested cluster is large, consisting of 32 genes in human comprising five subfamilies of Late Cornified Envelope genes (LCE1, LCE2, LCE3, LCE4, and LCE5), a cysteine-rich C-terminal gene, and 4 subfamilies of Small Proline-Rich proteins (SPPR1, SPRR2, SPRR3, SPRR4). The Small Proline-Rich proteins have diversified over time to provide tissue specific expression in different types of skin barriers [35]. There are only three interspersed genes within this complex and two of the three, involucrin and loricrin, are also expressed by keratinocytes within the Late Cornified Cell Envelope. Interestingly, these two genes were identified as genes whose position has potentially undergone selection for its proximity near interacting genes partners that exist within the cluster [36].

### Immunoglobulin Clusters

The immunoglobulin superfamily domain is one of the most versatile domains in the genome, leading to a variety of functions including cell-cell recognition, cell-surface receptors, muscle structure and, in higher organisms, the immune system [37]. This diversity emerges from the combinations and arrangements within genes of four subtypes of immunoglobulin domains: variable (V-set), constant-1 (C1-set), constant-2 (C2-set) and intermediate (I-set) [38] and paraclusters of the immunoglobulin superfamily consist of genes with highly variable combinations of these subtypes. Paraclusters of the immunoglobulin superfamily are particularly unique in their genomic arrangements, consisting of large clusters, up to 5 Mb in size, consisting of highly diversified family members with many unrelated genes interspersed among them. The diversity of these genes within these paraclusters is great enough to make detection of the paralogous relationships among them difficult when only using sequence similarity approaches; they are more readily detected using the domain datasets.

Human chromosome 19 is exceptional in the number of immunoglobulin paraclusters it contains, including a 2 Mb Siglec gene paracluster [39], a 1 Mb Leukocyte Receptor Complex [40], both of which are found in all mammals tested, and a 3 Mb immunoglobulin paracluster that appears in zebrafish as well that includes two subgroups, the Carcinoembryonic Antigen (CEA) gene family of cell adhesion molecules and a group of pregnancy-specific glycoproteins. Finally, a list of large immunoglobulin paraclusters must obviously include both the Major Histocompatibility Complex (MHC) on human chromosome 6 and an additional immune function cluster on human chromosome 1 with paralogous overlap to the MHC [41]. In the mouse, this latter paracluster has been shown to be under positive selection for particular combinations of alleles [42]. It is an ancient cluster, with origins common to the chicken MHC region and much smaller clusters in the fruit fly genome containing only a few genes. Overall around 20 immunoglobulin clusters were detected in humans all of which were positioned on chromosomes 1, 3, 6, 11, 17 and 19. There is good evidence according to Ensembl paralogy data that most of these paraclusters, including the MHC, are related to one or another, since each cluster contains paralogs of the others and all appear interconnected by the various paralogous gene groups being shared between them. Some clusters such as on 3, 11, and 19 are highly connected to one another and the others. This pattern of organization is consistent with their origin from ancient regions of duplicated genes that then diversified and dispersed across the genome into subclusters that underwent further diversification and expansions.

### Sequence similarity amongst genes in paraclusters

To better understand how these gene clustering results based on structural annotation data differ from the results of sequence similarity approaches, we compared human paraclusters with human datasets downloaded from the Duplicated Gene Database (DGD) which groups genes with sequence similarity by proximity with no applied measurement of statistical significance. There were 479 clusters reported by the DGD that were not detected as paraclusters because they were either too distantly spaced (>15 genes apart) or lacked statistical significance (>0.01 expectation cutoff). Additionally, there were 204 groups within DGD that contained additional genes not found within paraclusters, primarily as a result of additional genes positioned too distal for detection. Reversing the direction of comparison, we found 236 paraclusters not included in the DGD; 132 of these involved uncharacterized genes, most likely not included in the DGD datasets which are based on build 65 of Ensembl, and 22 clusters involved annotation errors. The remaining clusters were likely undetected in the DGD due to their low sequence similarity, since all but 8 pairs had no more than 35% sequence identity over 50% of the protein length in both directions. These clusters consisted of distinct classes of genes characterized by tight genomic clustering, the majority of which are tandemly arrayed, and highly sequence divergent. Cytokines (including interferons, chemokines and interleukins), cytokine receptors and chemokine-like factors predominate, but other classes found were claudins, complement factors, and apolipoproteins.

Additionally we found 229 paraclusters that included additional genes not found in the corresponding DGD groups. Of these, there were 45 cases which involved uncharacterized genes, 12 cases involving annotation errors, and 17 cases involving potential false positives where the “gravitional effect” may be occurring, i.e. inclusion of a gene distal to a large paracluster with which it shares only a single domain that is common in the genome. The remaining cases involved a large number of genes with low sequence similarity to other paracluster members and presumably were not found in DGD groups for this reason. These genes include cytokines, growth hormone/chorionic somatomammotropins, secretoglobins, FXYD domain containing ion transport regulators, apolipoproteins, immunoglobulins, defensins, olfactory receptors, tripartite motif-containing genes, lipocalins, ribonucleases, serine peptidase inhibitors, and C2H2 zinc fingers. In many cases involving these types of genes, multiple members were missing from individual DGD groups, suggesting a prevalence for sequence diversification within these paraclusters.

## Discussion

With the exception of *S. cerevisiae*, paraclusters are a prominent feature of the genomes of all the organisms we analyzed, including vertebrate, invertebrate and plant species. They are far from static on an evolutionary scale, preserving function despite considerable variation in their numbers, composition and identity. This variation is the product of differences in rates of gene duplication, neutral mutational drift and selection for favorable gene combinations. But whatever their evolutionary origin, the very existence of paraclusters is a functionally important aspect of contemporary genomes: genetically, in providing linked inheritance of functionally related genes, conceptually, in providing an evolutionary substrate for genomic specialization, and experimentally, in affecting our abilities to map quantitative trait loci.

Previous studies have reported on the presence of tandemly arrayed paralogs (TAGs) in a diverse set of species [5], [6], [7]. Where they overlap, our analyses are in agreement. However, from Figure 2 it is apparent that many paralogs are in proximity more frequently than what is expected by chance even when they are not in strictly tandem arrays because they are separated by interspersed, unrelated genes. This is particularly true of the paraclusters whose members share the immunoglobulin superfamily domain defined by SCOP and the immunoglobulin-like domain defined by InterPro. Our results in this case agree with previous work analyzing protein domain clustering in humans [43]. Clearly the paralogy of these genes long pre-dates the origins of the species in which they are found, and the origins of some highly diverse clusters likely represents once tandem duplication arrays that have since diversified, possibly involving domain shuffling, and rearranged with new genes interspersed. The true extent of these types of paraclusters go undetected in the TAG reports due to their sequence divergence and interspersed genes, but their clustering is in agreement with studies related to functional gene clustering. It is clear at this time that functional clustering is an important feature of contemporary genomes; what remains at issue is determining the identity and characteristics of the clusters and the evolutionary question of whether they arise from proximate duplications of genomic material versus the migration together of structurally unrelated genes. Several papers have reported on these questions, and a reexamination of their evidence suggests that a considerable majority of the clustering that has been observed is duplicative in origin. Lee and Sonnhammer [9] described clustering of KEGG pathway genes, but considered that two proteins were not structurally related unless they had both the same EC number and showed greater than 60% sequence identity, a quite stringent requirement. Two studies used shared GO (Gene Ontology) annotations as evidence of functional relatedness. Al Shahrour et al. [10] concluded that genomic duplications were not a factor in functional clustering based on the failure to find an excess of proteins meeting the very stringent requirement of 95 to 98% sequence similarity in clusters v. control regions. It was not surprising then that 97% of the genes containing their 5% most significant clusters in the human genome are also present in the paracluster dataset. In the case of Yi et al [44], who also used GO and also concluded that duplications are not a significant factor, 82% of the genes in their 5% most significant clusters again overlapped those in the paracluster dataset, suggesting that genomic duplications do play a significant role in functional clustering. This is not to say that all functional clustering is necessarily duplicative in origin, but it does suggest that further work will be required to begin resolving issues of how functional clusters arise in evolution.

Although small paraclusters containing only a very few genes predominate in the cluster counts throughout various species, large clusters, although less frequent, are as substantial in terms of the total number of genes they contain (Figure 2). The two groups differ significantly in their properties. Small paraclusters are likely to be of more recent evolutionary origin and large clusters are home to the most abundant protein domains. In humans, the Family G protein-coupled receptor-like, C2HC zinc finger, and Immunoglobulin domains, which collectively account for 30% of all functional domains found in human paraclusters, are found almost exclusively in paraclusters containing 10 or more genes. The evolutionary role of such highly abundant domains is emphasized in Nei's theory [45] that gene copy number, rather than gene count, is more highly correlated with organismal complexity. In this regard, Vogel has reported that C2H2 and C2HC zinc fingers, along with Immunoglobulin domains, have the highest abundance and correlate most strongly with the numbers of cell types found among 38 eukaryotes [46]. Expansion of these domains appears to have been particularly important in vertebrate evolution, providing novel genetic material on which selection has acted, andin mammals, expansions of C2H2 genes with KRAB domains, and G-protein coupled receptors (particularly olfactory receptors in rodents) appear to be important along with the continual expansion of immunoglobulin genes (Table 6).

**Table 6:** Most abundant superfamily domains in paraclusters.

family	hs	xp	ru	mm	rn	dg	bv	op	gg	da
Family A G protein-coupled receptor-like	379 (1)	280 (2)	322 (1)	1300 (1)	1229 (1)	709 (1)	377 (1)	916 (1)	49 (2)	145 (2)
C2H2 and C2HC zinc fingers	321 (2)	301 (1)	290 (2)	259 (2)	121 (3)	53 (6)	93 (3)	275 (3)	2 (126)	76 (5)
KRAB domain (Kruppel-associated box)	251 (3)	234 (3)	226 (4)	235 (3)	83 (4)	89 (3)	75 (4)	250 (4)	3 (101)	0
Immunoglobulin	202 (4)	190 (4)	239 (3)	212 (4)	351 (2)	275 (2)	177 (2)	283 (2)	78 (1)	168 (1)
Histone-fold	62 (5)	50 (9)	62 (5)	73 (8)	39 (14)	59 (4)	30 (12)	14 (30)	26 (4)	28 (16)
Intermediate filament protein, coiled coil region	57 (6)	56 (5)	48 (6)	57 (12)	48 (8)	55 (5)	47 (6)	35 (9)	12 (17)	22 (19)
Cadherin-like	56 (7)	53 (7)	31 (11)	46 (16)	47 (10)	46 (7)	21 (19)	37 (7)	29 (3)	47 (11)
Trypsin-like serine proteases	56 (8)	51 (8)	42 (8)	104 (5)	83 (5)	45 (8)	44 (7)	30 (14)	11 (20)	83 (3)
C-type lectin-like	52 (9)	53 (6)	44 (7)	86 (6)	65 (6)	35 (10)	47 (5)	31 (11)	18 (6)	57 (7)
Homeodomain-like	39 (10)	38 (10)	35 (10)	82 (7)	55 (7)	38 (9)	35 (9)	30 (13)	23 (5)	57 (8)
P-loop containing nucleoside triphosphate hydrolases	25 (19)	19 (27)	24 (16)	20 (33)	15 (38)	24 (15)	39 (8)	32 (10)	10 (23)	77 (4)
Periplasmic binding protein-like I	0	0	0	18 (43)	48 (9)	0	0	71 (5)	0	45 (12)

The top 5 superfamilies having the most paracluster genes were selected from each vertebrate species tested and the number of genes along with the superfamily rank is provided for each species. hs = human, xp = chimp, ru = macaque, mm = mouse, rn = rat, dg = dog, bv = cow, op = opossum, gg = chicken, da = zerbrafish.

For at least some paraclusters the role of purifying selection seems apparent. Among the various neurotransmitter receptors, considerable differences in the extent to which their genes have been maintained in paraclusters suggest that molecular interactions to form heteromeric functional molecules have played a significant role. Other examples of colocalization of heteromeric proteins include complement components, voltage dependant Ca+ channels, FXYD domain containing ion transport regulators, troponins, and the histone genes. In the case of the HOX paraclusters, maintenance of particular regulatory patterns seems to have been the factor; as first described in Drosophila, the order of the nine genes is co-linear with their action along the axis of the embryo. And we know experimentally that the chromosome 1 cluster of immunoglobulin genes is under purifying selection [42], presumably for its role in immune processes. The other pattern where purifying selection is apparent is the so-called multiple-variable-first exon pattern where tandem arrays of genes sharing a common set of 3′ exons are attached to variable 5′ exons with different promoter properties [47], [48]. This is exemplified by the protocadherins that also play a role in neurotransmission [49], [50]. Cell specific transcription is initiated by the promoter at one of the 5′ exons, which is then spliced to the same three constant exons, skipping over any intervening exons [51], [52]. Similar patterns are seen in UDP glucuronosysltransferase, plectin, neuronal nitric oxide synthase and glucocorticoid receptor genes [47].

As evidenced by the considerable differences among species and clades, paracluster evolution is dramatic, with constant creation of new clusters, expansions of existing clusters, and dissolution of old ones, to the extent that many genes within paraclusters as well as whole paraclusters appear species specific at the level of analysis now possible. What is interesting in this regard is the common observation that it is the demands of cell-to-cell communication and environmental interactions, especially the presence of infectious agents and xenobiotics, which often appears to drive paracluster expansion exemplified by the expansion of ZNF clusters in defense against proliferation of retroviral LTRs [53]. The genome of the microcrustacean, *Daphnia pulex*, is a prime example as the expression of paralogous genes, many of which are in tandem arrays, is associated with environmental perturbations [54]. In turn, this raises the question of the extent to which formation of novel paraclusters contributes to the process of speciation itself by facilitating niche adaptations.

## Materials and Methods

To quantify the numbers of genes sharing structural annotation data that are in proximity of one another, a chromosome walk algorithm was developed that evaluates each gene in succession, comparing its structural annotation data to each of the neighboring genes up to n genes away, where n represents the span in the forward direction from the target gene (in the case when n = 2, the two genes are adjacent). In this analysis we looked at all spans up to n = 100. A separate chromosome walk was performed for each of the five datasets (InterPro, SCOP, PANTHER, Ensembl families, and Ensembl paralogs) and for each gene pair we tested propositions relevant to the specific annotation dataset such as “do you share an InterPro domain”, “do you share a SCOP superfamily domain”, “are you paralogous according to Ensembl”, “do you belong to the same Ensembl protein family”, and “do you belong to the same PANTHER superfamily”. Genome wide metrics were obtained in this way for each species. To control for random effects, randomized genomes were created by permuting a given gene order without disrupting gene annotations. Averaging five permuted genomes created a smooth frequency distribution curve of positive gene pairs across all the spans tested, forming a straight line almost flat with a very slight negative slope caused by the limiting size of individual chromosomes (Figure 1a).

Gene clusters were qualitatively identified as a product of chromosome walks by chaining together genes that are in proximity to one another and share a common structural annotation according to one of the five datasets tested. Each dataset was tested separately. The chaining algorithm was implemented in a forward direction along the chromosomes and each gene was evaluated for inclusion in no more than one chain. Although the genes in a chain can share multiple combinations of annotations, the chain itself is represented by the least common subset of annotations shared by all its members. A new gene is added to a chain only if it shares at least one of the common annotations; if it does not share all of the common annotations, its inclusion will reduce the least common subset of annotations accordingly. Because chains are constructed by moving in rank order along a chromosome, the combination of domains represented within a chain could be influenced by gene ordering.

Knowing the frequency in each genome of all the possible annotation values in each of the five datasets, a p-value was obtained for each putative chain by applying the hypergeometric probability distribution. For a given chain consisting of *k* genes (counting interstitial genes), the probability that at least *l* genes share one or more annotation values is given by
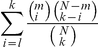
where *N* is the number of genes in the genome and *m* is the number of genes in the genome that share the annotation value(s).

P-values were subsequently corrected for according to the number of possible chains given the sizes of both the chain and the relevant genome; this provided a random expectation value for a given chain based on the size of the genome in consideration. Two parameters of the chaining algorithm were tested empirically, the allowed size of a gap (number of consecutive genes which do not share in the annotation), and the total number of gaps allowed in a cluster. It was determined that limiting the total number of gaps was not critical to the total amount of clustering and was only done so to manage the computer runtime performance. Changing the size of allowed gaps did however have an impact on the results, but only when incrementing at smaller values. Varying this parameter from 0 to 1 varied the total number of clustered genes between 1 and 3 percent within the various species and this effect diminished as the parameter was increased. An effect of less than a tenth of a percent was obtained for all species by varying the gap length from 14 to 15, so a gap length of 15 is the value we chose to implement as a threshold across all species. Nested tandem arrays of a different family were counted as only one gap space in this analysis.

**Figure 6:**
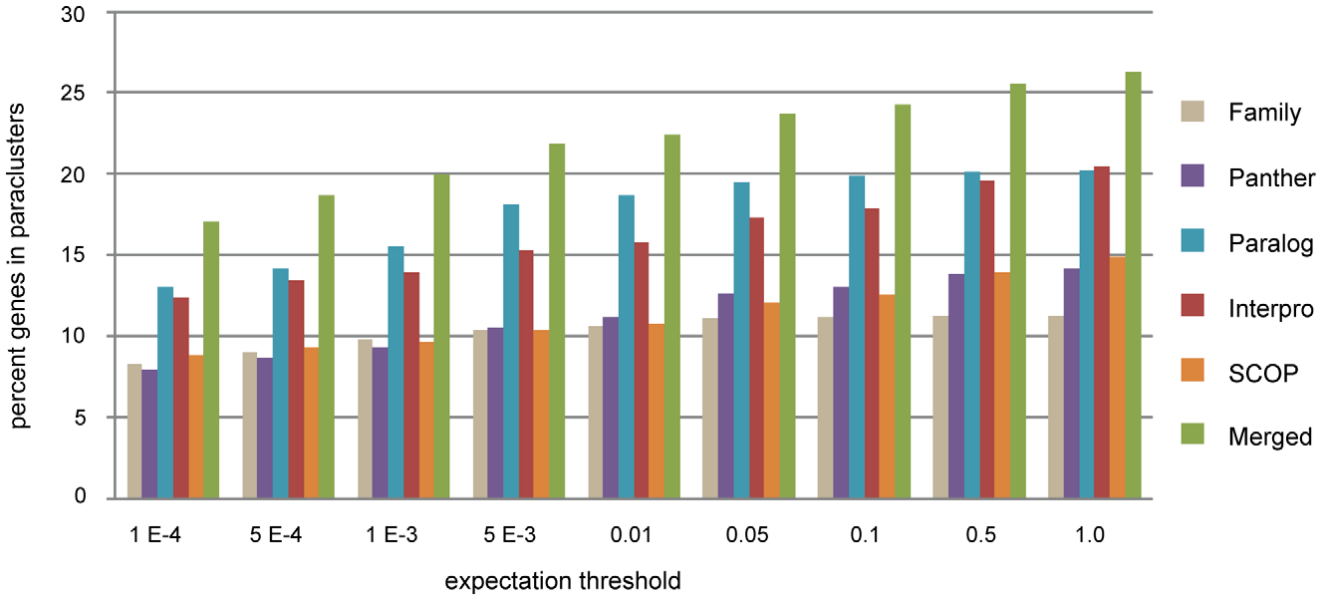
Effect of expectation threshold on total clustering metrics. The number of genes included within paraclusters as a function of the choice of expectation threshold shown for each dataset along with all datasets merged.

We evaluated the total amount of clustering as a function of the stringency of the expectation threshold required for selecting a chain as significant by performing multiple chromosome walks and applying various expectation thresholds in both the real and randomly permuted genomes. Empirically we found that requiring e<0.01 practically eliminated the detection of gene clusters among randomized genomes in all the annotation systems tested across all species. This provided a quite conservative threshold for paracluster detection, with a low probability of false positive clusters, and resulting in a minimum estimation of genome wide paraclustering metrics. Furthermore, at the e<0.01 threshold, essentially all clusters greater than two genes were identified. When evaluating higher thresholds the tendency in cluster identification was to include additional clusters containing only two genes having increasing space between them. Empirically we found that those datasets which involved whole gene annotation (Ensembl family, Ensembl paralogy, and PANTHER) leveled off in total clustering as the threshold was increased to e<0.1 and greater. Any increase in the total genome wide clustering metrics above this threshold was almost all due to the domain specific annotation datasets, namely InterPro and SCOP (Figure 6).

Because this method is dependent on the quality and extensiveness of annotation data for each of the studied genomes, we evaluated the impact of genes in a given species having no annotations in any of the datasets, consequently creating dark regions in the genome according to the methodology. Actually, for each species we obtained 100 percent annotation coverage simply due to the inclusion of the Ensembl family annotations which involve all genes, but we investigated whether eliminating Ensembl family annotations from the analysis otherwise revealed large annotation gaps. Doing this, we found that some genomes had less coverage than others; for example the chicken genome and the fly genome, having the smallest coverage, had only around 86% of mapped genes in total coverage whereas humans had 93%. But we were able to conclude based on specific characteristics of these regions that their contribution to total paraclustering would be small and their impact would be inconsequential to the total reported metrics. In particular, all these genes have no reported paralogs and where orthologs of these genes were found in human, these orthologs rarely shared any paralogy with other human genes and only rarely belonged to any of the detected human paraclusters (for example, less than 0.1% of all chicken genes were found to fit this description and only 0.03% of fly genes do so). And furthermore, in both chicken and fly the amount of clustering detected based on the Ensembl family dataset for genes within these regions was very small (0.1% of chicken genes and 0.3% of fly genes).

Genome data from build 58 of Ensembl was downloaded utilizing the Biomart interface for the following organisms and their corresponding assemblies: human (GRCh37), chimp (CHIMP2.1), macaque (MMUL_1.0), mouse (NCBIM37), rat (RGSC3.4), cow (Btau_4.0), dog (CanFam_2.0), opossum (monDom5), chicken (WASHUC2), zebrafish (Zv8), yeast (SGD1.01), fly (BDGP5.13), worm (WS210), and Arabidopsis (TAIR9). The gene annotation data downloaded from the PANTHER website was based on version 7.0 of the PANTHER HMM library. The data downloaded from SCOP Superfamily was dated May 30, 2010, the InterPro data was version 26, dated March 24, 2010, and the Inparanoid data was from version 7.0, updated June 2009.

## Supporting Information

Table S1 **List of species analyzed along with their corresponding number of protein coding genes, number of chromosomes, and total length of chromosomes according to Ensembl build 58.**
(DOC)

Table S2 **Total number of genes detected within paraclusters by merging results from all datasets and the number of paracluster genes detected in common between any two datasets.**
(DOC)

Table S3 **Characteristics of paraclusters detected exclusively by SCOP and/or InterPro databases.**
(DOC)

Table S4 **The count of genes found in paraclusters and the number of paraclusters as detected by each of the five datasets (Ensembl family, Ensembl paralogy, SCOP Superfamily, InterPro, and PANTHER) and all datasets combined for all species analyzed.**
(DOC)

Table S5 **A comparison by species of the most abundant SCOP superfamily domains represented within paraclusters.**
(DOC)
