# Supplementary material for: Clusters of Ancestrally Related Genes That Show Paralogy in Whole or in Part Are a Major Feature of the Genomes of Humans and Other Species
Source: PLoS One. 2012 Apr 26;7(4):e35274. doi: 10.1371/journal.pone.0035274 (PMC3338513; doi:10.1371/journal.pone.0035274)
Supplement: Table S1 — List of species analyzed along with their corresponding number of protein coding genes, number of chromosomes, and total length of chromosomes according to Ensembl build 58. (DOC) [file pone.0035274.s001.doc]

**Table S1. Genome metrics for each species.**

| **species** | **genes** | **# chromosomes** | **chr length (Gb)** | **assembly** |
| --- | --- | --- | --- | --- |
| Homo sapiens | 20,686 | 24 | 3.1 | GRCh37 |
| Pan troglodytes | 19,199 | 25 | 3.35 | CHIMP2.1 |
| Macaca mulatta | 21,023 | 21 | 3.1 | MMUL_1.0 |
| Mus musculus | 22,793 | 21 | 2.72 | NCBIM37 |
| Rattus norvegicus | 22,925 | 21 | 2.72 | RGSC3.4 |
| Canis familiaris | 19,014 | 39 | 2.53 | CanFam_2.0 |
| Bos taurus | 19,030 | 30 | 2.92 | Btau_4.0 |
| Monodelphis domestica | 18,640 | 9 | 3.61 | monDom5 |
| Gallus gallus | 15,310 | 31 | 1.1 | WASHUC2 |
| Danio rerio | 22,940 | 25 | 1.48 | Zv8 |
| Drosophila melanogaster | 13,858 | 7 | 0.17 | BDGP5.13 |
| Caenorhabditis elegans | 20,212 | 6 | 0.1 | WS210 |
| Saccharomyces cerevisiae | 6,666 | 16 | 0.01 | SGD1.01 |
| Arabidopsis thaliana | 31,070 | 5 | 0.12 | TAIR9 |
