# Supplementary material for: Clusters of Ancestrally Related Genes That Show Paralogy in Whole or in Part Are a Major Feature of the Genomes of Humans and Other Species
Source: PLoS One. 2012 Apr 26;7(4):e35274. doi: 10.1371/journal.pone.0035274 (PMC3338513; doi:10.1371/journal.pone.0035274)
Supplement: Table S2 — Total number of genes detected within paraclusters by merging results from all datasets and the number of paracluster genes detected in common between any two datasets. (DOC) [file pone.0035274.s002.doc]

**Table S2. Counts of paracluster genes found in common between datasets for each species.**

| **Homo sapiens** | Ensembl  paralog | Ensembl  family | PANTHER | SCOP | InterPro |  |
| --- | --- | --- | --- | --- | --- | --- |
| 4638 | 3861 | 2041 | 2052 | 1806 | 2712 | Ensembl paralog |
|  |  | 2195 | 1018 | 1138 | 1599 | Ensembl family |
|  |  |  | 2311 | 1319 | 1788 | PANTHER |
|  |  |  |  | 2227 | 2063 | SCOP |
|  |  |  |  |  | 3269 | InterPro |
| **Pan troglodytes** | paralog | family | PANTHER | SCOP | InterPro |  |
| 3622 | 2819 | 1578 | 1913 | 1522 | 2094 | Ensembl paralog |
|  |  | 1783 | 1115 | 1072 | 1351 | Ensembl family |
|  |  |  | 2211 | 1294 | 1782 | PANTHER |
|  |  |  |  | 1939 | 1853 | SCOP |
|  |  |  |  |  | 2643 | InterPro |
| **Macaca mulatta** | paralog | family | PANTHER | SCOP | InterPro |  |
| 4090 | 3212 | 1777 | 1962 | 1508 | 2020 | Ensembl paralog |
|  |  | 2073 | 1136 | 1017 | 1291 | Ensembl family |
|  |  |  | 2318 | 1237 | 1660 | PANTHER |
|  |  |  |  | 1951 | 1841 | SCOP |
|  |  |  |  |  | 2544 | InterPro |
| **Mus musculus** | paralog | family | PANTHER | SCOP | InterPro |  |
| 5887 | 5074 | 3493 | 2641 | 3259 | 3946 | Ensembl paralog |
|  |  | 3689 | 1652 | 2634 | 3003 | Ensembl family |
|  |  |  | 2951 | 1892 | 2353 | PANTHER |
|  |  |  |  | 3696 | 3494 | SCOP |
|  |  |  |  |  | 4497 | InterPro |
| **Rattus norvegicus** | paralog | family | PANTHER | SCOP | InterPro |  |
| 5930 | 5112 | 3505 | 1881 | 2718 | 3964 | Ensembl paralog |
|  |  | 3754 | 1276 | 2219 | 3010 | Ensembl family |
|  |  |  | 2089 | 1134 | 1679 | PANTHER |
|  |  |  |  | 3077 | 2918 | SCOP |
|  |  |  |  |  | 4514 | InterPro |
| **Bos taurus** | paralog | family | PANTHER | SCOP | InterPro |  |
| 4450 | 3655 | 2497 | 2206 | 1478 | 2582 | Ensembl paralog |
|  |  | 2767 | 1469 | 1103 | 1951 | Ensembl family |
|  |  |  | 2514 | 1156 | 1901 | PANTHER |
|  |  |  |  | 1798 | 1667 | SCOP |
|  |  |  |  |  | 3069 | InterPro |
| **Canis familiaris** | paralog | family | PANTHER | SCOP | InterPro |  |
| 4070 | 3258 | 2147 | 1759 | 1713 | 2154 | Ensembl paralog |
|  |  | 2372 | 1068 | 1293 | 1606 | Ensembl family |
|  |  |  | 2054 | 1116 | 1446 | PANTHER |
|  |  |  |  | 2129 | 1978 | SCOP |
|  |  |  |  |  | 2663 | InterPro |
| **Monodelphis domestica** | paralog | family | PANTHER | SCOP | InterPro |  |
| 4161 | 3386 | 2468 | 1842 | 2114 | 2580 | Ensembl paralog |
|  |  | 2795 | 1267 | 1870 | 2165 | Ensembl family |
|  |  |  | 2091 | 1281 | 1654 | PANTHER |
|  |  |  |  | 2556 | 2448 | SCOP |
|  |  |  |  |  | 3095 | InterPro |
| **Gallus gallus** | paralog | family | PANTHER | SCOP | InterPro |  |
| 1962 | 1517 | 803 | 649 | 562 | 967 | Ensembl paralog |
|  |  | 909 | 376 | 359 | 566 | Ensembl family |
|  |  |  | 781 | 393 | 577 | PANTHER |
|  |  |  |  | 730 | 693 | SCOP |
|  |  |  |  |  | 1246 | InterPro |
| **Danio rerio** | paralog | family | PANTHER | SCOP | InterPro |  |
| 5820 | 4853 | 3545 | 589 | 1345 | 2897 | Ensembl paralog |
|  |  | 4126 | 486 | 1129 | 2458 | Ensembl family |
|  |  |  | 626 | 390 | 491 | PANTHER |
|  |  |  |  | 1594 | 1383 | SCOP |
|  |  |  |  |  | 3420 | InterPro |
| **Drosophila melanogaster** | paralog | family | PANTHER | SCOP | InterPro |  |
| 3088 | 2543 | 1358 | 1453 | 914 | 1652 | Ensembl paralog |
|  |  | 1624 | 934 | 638 | 1037 | Ensembl family |
|  |  |  | 1590 | 727 | 1231 | PANTHER |
|  |  |  |  | 1023 | 963 | SCOP |
|  |  |  |  |  | 1891 | InterPro |
| **Saccharomyces cerevisiae** | paralog | family | PANTHER | SCOP | InterPro |  |
| 217 | 142 | 90 | 34 | 26 | 51 | Ensembl paralog |
|  |  | 148 | 33 | 22 | 72 | Ensembl family |
|  |  |  | 44 | 18 | 30 | PANTHER |
|  |  |  |  | 46 | 36 | SCOP |
|  |  |  |  |  | 98 | InterPro |
| **Caenorhabditis elegans** | paralog | family | PANTHER | SCOP | InterPro |  |
| 5213 | 4,094 | 2,593 | 2,237 | 1,142 | 1,846 | Ensembl paralog |
|  |  | 3,128 | 1,807 | 980 | 1,379 | Ensembl family |
|  |  |  | 2,647 | 1,080 | 1,576 | PANTHER |
|  |  |  |  | 1,474 | 935 | SCOP |
|  |  |  |  |  | 2,160 | InterPro |
| **Arabidopsis thaliana** | paralog | family | PANTHER | SCOP | InterPro |  |
| 5000 | 4,352 | 535 | 1,579 | 1,514 | 2,465 | Ensembl paralog |
|  |  | 577 | 299 | 425 | 511 | Ensembl family |
|  |  |  | 1,782 | 903 | 1,310 | PANTHER |
|  |  |  |  | 1,668 | 1,512 | SCOP |
|  |  |  |  |  | 2,880 | InterPro |
