# Supplementary material for: Clusters of Ancestrally Related Genes That Show Paralogy in Whole or in Part Are a Major Feature of the Genomes of Humans and Other Species
Source: PLoS One. 2012 Apr 26;7(4):e35274. doi: 10.1371/journal.pone.0035274 (PMC3338513; doi:10.1371/journal.pone.0035274)
Supplement: Table S3 — Characteristics of paraclusters detected exclusively by SCOP and/or InterPro databases. (DOC) [file pone.0035274.s003.doc]

**Table S3. Characteristics of paraclusters detected exclusively by SCOP and/or InterPro databases.**

| **paracluster** | **related paracluster** | **size** | **homology type** | **paralog test** | **ortholog test** | **ancestor** | **%id %length (target)**  **%id %length (query)** |
| --- | --- | --- | --- | --- | --- | --- | --- |
| BCL2L13, BID |  | 2,2 | complete | n | n | Tetrapoda | 0 0 0 0 * |
| CSN1S1, CSN2 |  | 2,2 | complete | n | n | Eutheria | 23 58 23 47 |
| ERG, ETS2 | ETS1, FLI1 | 2,2 | paralogs | y | y | Euteleostomi | 34 55 34 74 |
| ETS1, FLI1 | ERG, ETS2 | 2,2 | paralogs | y | y | Euteleostomi | 47 30 48 34 |
| HTR4, ADRB2 | DRD1, HRH2 | 2,2 | paralogs | y | y | Euteleostomi | 36 75 36 77 |
| KCNJ1, KCNJ5 | KCNJ10, KCNJ9  KCNA7, TRPM4 | 2,2 | paralogs | y | y | Euteleostomi | 45 89 45 82 |
| KCNJ10, KCNJ9 | KCNJ1, KCNJ5  KCNA7, TRPM4 | 2,2 | paralogs | y | y | Euteleostomi | 41 90 41 88 |
| LIF, OSM | LIFR, OSMR | 2,2 | complete | n | n | Euteleostomi | 32 15 32 12 |
| LMOD2, WASL |  | 2,2 | joined | n | n | Tetrapoda | 29 24 29 30 |
| PRNP, PRND, PRNT |  | 2,2 | complete | n | n | Theria | 24 61 25 80 |
| PRRG1, RP5-972B16.2 |  | 2,2 | fused | n | n | Human | 100 26 100 20 |
| PTPN4, EPB41L5 |  | 2,2 | joined | n | n | Euteleostomi | 40 36 40 46 |
| SFTPB, GNLY |  | 2,2 | complete | n | n | Boreoeutheria | 18 23 18 66 |
| TNNT1, TNNI3 | TNNT2, TNNI1  TNNI2, TNNT3 | 2,2 | complete | y | y | Metazoa | 23 51 23 62 |
| DRD1, HRH2 | HTR4, ADRB2 | 2,3 | paralogs | y | y | Euteleostomi | 34 82 34 82 |
| TNNT2, TNNI1 | TNNT1, TNNI3  TNNI2, TNNT3 | 2,3 | complete | y | y | Metazoa | 25 36 25 54 |
| TNNI2, TNNT3 | TNNT1, TNNI3  TNNT2, TNNI1 | 2,4 | complete | y | y | Vertebrata | 19 53 19 35 |
| KCNE2, KCNE1 |  | 2,5 | complete | n | y | Euteleostomi | 45 40 45 39 |
| KCNA7, TRPM4 | KCNJ1, KCNJ5  KCNJ10, KCNJ9 | 2,6 | mixed | y | y | Gnathostomata | 22 24 22 8 |
| PPP5C, AC011484.2 |  | 2,6 | partial | n | n | Catarrhini | 93 18 93 54 |
| CPS1, ATIC |  | 2,8 | mixed | y | y | Amniota | 22 12 22 32 |
| CCL22, CX3CL1, CCL17 |  | 3,3 | complete | n | n | Theria | 30 64 30 66 |
| DCDC5, DCDC1 |  | 3,3 | partial | n | n | Amniota | 31 10 31 23 |
| FASLG, TNFSF18, TNFSF4 | TNFSF9, CD70, TNFSF14 | 3,3 | complete | n | n | Tetrapoda, Mammalia | 23 46 23 64 * |
| HIST3H3, HIST3H2A, HIST3H2BB |  | 3,3 | complete | y | y | Mammalian | 20 55 20 57 |
| IFNG, IL26, IL22 | IL20RA, IL22RA2, IFNGR | 3,3 | complete | n | n | Tetrapoda | 24 59 24 58 * |
| TNFSF9, CD70, TNFSF14 | FASLG,TNFSF18, TNFSF4 | 3,3 | complete | n | n | Tetrapoda | 29 50 29 50 |
| COL5A1, FCN2, FCN1 |  | 3,4 | joined | n | n | Theria | 53 21 53 31 |
| DMBT1, CUZD1 |  | 3,4 | joined | n | n | Amniota | 38 15 38 64 |
| LRRN2, NFASC, CNTN2, TMEM81 | CHL1, CNTN6, CNTN4, IL5RA, LRRN1 | 4,4 | paralogs, mixed, partial | y | y | Vertebrata | 29 21 29 32 * |
| FXYD3, FXYD1, FXYD7, FXYD5 | FXYD2, FXYD6 | 4,5 | complete | n | n | Amniota | 46 39 46 38 |
| IL3, CSF2, IL5, IL13, IL4 | CRLF2, CSF2RA, IL3RA | 5,12 | complete | n | n | Theria | 29 36 29 38 * |

The ‘related paracluster’ column represents a paracluster that is related to the first (mostly based on structural evidence yet some cases are based on functional relatedness where the two clusters possess ligand and receptor genes corresponding to one another); the ‘size’ column contains two values representing the number of genes in the paracluster excluding and including interstitial genes; the ‘type’ column describes the type of paracluster classified in one of the following ways: ‘paralogs’ indicates that the genes became annotated as paralogous in a subsequent build to which we based our datasets on (build 58); ‘complete’ indicates that the two genes share consistent domain architectures across the entire length of the genes, allowing for any one domain to repeat; ‘partial’ is where one gene’s complete domain architecture is only a partial match to other members of the cluster; ‘joined’ indicates that a gene acquires a domain from the cluster evidenced by the observation that no other paralog of the gene that exists elsewhere in the genome possesses the acquired domain; ‘fused’ indicates that a gene is a combination of domains of the two unrelated neighboring genes; the ‘ortholog test’ and ‘paralog test’ columns represent indicators of whether or not an ortholog or a paralog was found whose speciation or duplication event predates the common ancestor of the paracluster; the ‘ancestor’ column represents the last common ancestor where the cluster could be detected; the ‘%id %length’ column represents the percent identity and percent length of both target and query genes within the paracluster (arbitrarily assigning the target and the query ), or the average of such values if more than two genes exist in the paracluster.

^*^ Some pair wise sequence comparisons had no significant sequence match and were left out of the averaging
