# Supplementary material for: Clusters of Ancestrally Related Genes That Show Paralogy in Whole or in Part Are a Major Feature of the Genomes of Humans and Other Species
Source: PLoS One. 2012 Apr 26;7(4):e35274. doi: 10.1371/journal.pone.0035274 (PMC3338513; doi:10.1371/journal.pone.0035274)
Supplement: Table S4 — The count of genes found in paraclusters and the number of paraclusters as detected by each of the five datasets (Ensembl family, Ensembl paralogy, SCOP Superfamily, InterPro, and PANTHER) and all datasets combined for all species analyzed. (DOC) [file pone.0035274.s004.doc]

**Table S4. Counts of paracluster genes found from each database for each species.**

| **species** | **database** | **genes annotated** | **genes in paraclusters** | **paracluster counts** | **percent genes in paraclusters** |
| --- | --- | --- | --- | --- | --- |
| Homo sapiens | Ensembl paralog | 14,827 | 3,861 | 1,162 | 18.7 |
|  | Ensembl family | 20,665 | 2,195 | 648 | 10.6 |
|  | PANTHER | 16,487 | 2,311 | 629 | 11.2 |
|  | SCOP | 13,507 | 2,227 | 345 | 10.8 |
|  | InterPro | 17,274 | 3,269 | 660 | 15.8 |
|  | merged | 20,686 | 4,638 | 1,133 | 22.4 |
| Pan troglodytes | Ensembl paralog | 13,366 | 2,819 | 894 | 14.7 |
|  | Ensembl family | 19,199 | 1,783 | 554 | 9.3 |
|  | PANTHER | 16,263 | 2,211 | 605 | 11.5 |
|  | SCOP | 12,803 | 1,939 | 308 | 10.1 |
|  | InterPro | 16,091 | 2,643 | 550 | 13.8 |
|  | merged | 19,199 | 3,622 | 925 | 18.8 |
| Macaca mulatta | Ensembl paralog | 15,788 | 3,212 | 1,100 | 15.3 |
|  | Ensembl family | 21,023 | 2,073 | 705 | 9.9 |
|  | PANTHER | 18,074 | 2,318 | 668 | 11.0 |
|  | SCOP | 14,009 | 1,951 | 307 | 9.3 |
|  | InterPro | 17,288 | 2,544 | 540 | 12.1 |
|  | merged | 21,023 | 4,090 | 1,125 | 19.4 |
| Mus musculus | Ensembl paralog | 16,057 | 5,074 | 1,149 | 22.3 |
|  | Ensembl family | 22,792 | 3,689 | 755 | 16.2 |
|  | PANTHER | 17,023 | 2,951 | 713 | 13.0 |
|  | SCOP | 15,267 | 3,696 | 391 | 16.2 |
|  | InterPro | 18,478 | 4,497 | 646 | 19.7 |
|  | merged | 22,793 | 5,887 | 1,100 | 25.8 |
| Rattus norvegicus | Ensembl paralog | 17,402 | 5,112 | 1,197 | 22.3 |
|  | Ensembl family | 22,925 | 3,754 | 827 | 16.4 |
|  | PANTHER | 14,113 | 2,089 | 544 | 9.1 |
|  | SCOP | 14,467 | 3,077 | 351 | 13.4 |
|  | InterPro | 19,365 | 4,514 | 657 | 19.7 |
|  | merged | 22,925 | 5,930 | 1,160 | 25.8 |
| Canis familiaris | Ensembl paralog | 13,985 | 3,258 | 1,065 | 17.1 |
|  | Ensembl family | 19,014 | 2,372 | 744 | 12.5 |
|  | PANTHER | 16,343 | 2,054 | 639 | 10.8 |
|  | SCOP | 13,627 | 2,129 | 302 | 11.2 |
|  | InterPro | 15,440 | 2,663 | 530 | 14.0 |
|  | merged | 19,014 | 4,070 | 1,111 | 21.4 |
| Bos taurus | Ensembl paralog | 14,225 | 3,655 | 1,105 | 19.2 |
|  | Ensembl family | 19,030 | 2,767 | 807 | 14.5 |
|  | PANTHER | 16,341 | 2,514 | 700 | 13.2 |
|  | SCOP | 12,315 | 1,798 | 312 | 9.4 |
|  | InterPro | 16,372 | 3,069 | 547 | 16.1 |
|  | merged | 19,030 | 4,450 | 1,120 | 23.3 |
| Monodelphis domestica | Ensembl paralog | 13,976 | 3,386 | 955 | 18.2 |
|  | Ensembl family | 18,640 | 2,795 | 700 | 15.0 |
|  | PANTHER | 15,802 | 2,091 | 590 | 11.2 |
|  | SCOP | 13,668 | 2,556 | 319 | 13.7 |
|  | InterPro | 16,023 | 3,095 | 538 | 16.6 |
|  | merged | 18,640 | 4,161 | 970 | 22.3 |
| Gallus gallus | Ensembl paralog | 9,451 | 1,517 | 584 | 9.91 |
|  | Ensembl family | 15,310 | 909 | 369 | 5.94 |
|  | PANTHER | 10,235 | 781 | 293 | 5.1 |
|  | SCOP | 9,680 | 730 | 188 | 4.8 |
|  | InterPro | 11,896 | 1,246 | 350 | 8.1 |
|  | merged | 15,310 | 1,962 | 669 | 12.8 |
| Danio rerio | Ensembl paralog | 18,902 | 4,853 | 1,583 | 21.2 |
|  | Ensembl family | 22,940 | 4,126 | 1,412 | 18.0 |
|  | PANTHER | 5,954 | 626 | 179 | 2.7 |
|  | SCOP | 13,564 | 1,594 | 297 | 7.0 |
|  | InterPro | 19,948 | 3,420 | 761 | 14.9 |
|  | merged | 22,940 | 5,820 | 1,734 | 25.3 |
| Caenorhabditis elegans | Ensembl paralog | 12,498 | 4,094 | 1,230 | 20.3 |
|  | Ensembl family | 20,212 | 3,128 | 1,088 | 15.5 |
|  | PANTHER | 12,807 | 2,647 | 588 | 13.1 |
|  | SCOP | 9,191 | 1,474 | 239 | 7.3 |
|  | InterPro | 13,480 | 2,160 | 469 | 10.7 |
|  | merged | 20,212 | 5,213 | 1,423 | 25.7 |
| Drosophila melanogaster | Ensembl paralog | 7,698 | 2,543 | 835 | 18.4 |
|  | Ensembl family | 13,858 | 1,624 | 595 | 11.7 |
|  | PANTHER | 9,549 | 1,590 | 493 | 11.5 |
|  | SCOP | 7,653 | 1,023 | 238 | 7.4 |
|  | InterPro | 10,695 | 1,891 | 481 | 13.7 |
|  | merged | 13,858 | 3,088 | 976 | 22.2 |
| Saccharomyces cerevisiae | Ensembl paralog | 2,025 | 142 | 60 | 2.1 |
|  | Ensembl family | 6,666 | 148 | 59 | 2.2 |
|  | PANTHER | 3,752 | 44 | 19 | 0.7 |
|  | SCOP | 3,499 | 46 | 19 | 0.7 |
|  | InterPro | 4,699 | 98 | 34 | 1.5 |
|  | merged | 6,666 | 217 | 85 | 3.2 |
| Arabidopsis thaliana | Ensembl paralog | 25,356 | 4,352 | 1,474 | 14.0 |
|  | Ensembl family | 6,674 | 577 | 135 | 1.9 |
|  | PANTHER | 14,825 | 1,782 | 561 | 5.7 |
|  | SCOP | 16,002 | 1,668 | 391 | 5.4 |
|  | InterPro | 23,673 | 2,880 | 762 | 9.3 |
|  | merged | 31,070 | 5,000 | 1,569 | 16.0 |
