# Supplementary material for: Clusters of Ancestrally Related Genes That Show Paralogy in Whole or in Part Are a Major Feature of the Genomes of Humans and Other Species
Source: PLoS One. 2012 Apr 26;7(4):e35274. doi: 10.1371/journal.pone.0035274 (PMC3338513; doi:10.1371/journal.pone.0035274)
Supplement: Table S5 — A comparison by species of the most abundant SCOP superfamily domains represented within paraclusters. (DOC) [file pone.0035274.s005.doc]

**Table S5. Most abundant superfamilies found within paraclusters for different species.**

| **H. sapiens** |  | **M. musculus** |  |
| --- | --- | --- | --- |
| Family A G protein-coupled receptor-like | 378 | Family A G protein-coupled receptor-like | 1300 |
| C2H2 and C2HC zinc fingers | 274 | C2H2 and C2HC zinc fingers | 220 |
| Immunoglobulin | 196 | Immunoglobulin | 185 |
| KRAB domain (Kruppel-associated box) | 91 | Trypsin-like serine proteases | 101 |
| Histone-fold | 62 | KRAB domain (Kruppel-associated box) | 86 |
| Intermediate filament protein | 57 | C-type lectin-like | 84 |
| coiled coil region | 57 | Homeodomain-like | 82 |
| Cadherin-like | 55 | Histone-fold | 73 |
| Trypsin-like serine proteases | 51 | RNI-like | 68 |
| C-type lectin-like | 50 | Cytochrome P450 | 66 |
| Homeodomain-like | 39 | 4-helical cytokines | 64 |
| Interleukin 8-like chemokines | 36 | Intermediate filament protein | 57 |
| 4-helical cytokines | 29 | coiled coil region | 57 |
| Cytochrome P450 | 29 | Lipocalins | 53 |
| EF-hand | 28 | alpha/beta-Hydrolases | 53 |
| **G. gallus** |  | **D. rerio** |  |
| Immunoglobulin | 71 | Immunoglobulin | 162 |
| Family A G protein-coupled receptor-like | 49 | Family A G protein-coupled receptor-like | 145 |
| Cadherin-like | 29 | Trypsin-like serine proteases | 82 |
| Histone-fold | 26 | C2H2 and C2HC zinc fingers | 76 |
| Homeodomain-like | 23 | P-loop containing nucleoside triphosphate hydrolases | 73 |
| C-type lectin-like | 18 | Protein kinase-like (PK-like) | 69 |
| Neurotransmitter-gated ion-channel transmembrane pore | 17 | Homeodomain-like | 57 |
| Nicotinic receptor ligand binding domain-like | 17 | C-type lectin-like | 56 |
| Cytochrome P450 | 16 | RING/U-box | 52 |
| Serpins | 16 | Cadherin-like | 47 |
| Complement control module/SCR domain | 13 | Periplasmic binding protein-like I | 45 |
| NAD(P)-binding Rossmann-fold domains | 13 | Cytochrome P450 | 41 |
| Interleukin 8-like chemokines | 12 | B-box zinc-binding domain | 35 |
| Intermediate filament protein | 12 | Concanavalin A-like lectins/glucanases | 34 |
| MFS general substrate transporter | 12 | RNI-like | 30 |
| **D. melanogaster** |  | **C. elegans** |  |
| Histone-fold | 88 | Family A G protein-coupled receptor-like | 425 |
| Trypsin-like serine proteases | 82 | Nuclear receptor ligand-binding domain | 105 |
| C-terminal domain | 45 | C-type lectin-like | 100 |
| Invertebrate chitin-binding proteins | 41 | Glucocorticoid receptor-like (DNA-binding domain) | 79 |
| alpha/beta-Hydrolases | 41 | Histone-fold | 61 |
| MFS general substrate transporter | 30 | TRAF domain-like | 59 |
| Cytochrome P450 | 29 | POZ domain | 45 |
| NAD(P)-binding Rossmann-fold domains | 29 | Cytochrome P450 | 36 |
| Thioredoxin-like | 28 | PapD-like | 35 |
| C2H2 and C2HC zinc fingers | 26 | C-terminal domain | 31 |
| Glutathione S-transferase (GST) | 26 | L domain-like | 30 |
| Zn-dependent exopeptidases | 26 | Glutathione S-transferase (GST) | 29 |
| Protein kinase-like (PK-like) | 24 | Thioredoxin-like | 29 |
| Metalloproteases ("zincins") | 23 | alpha/beta-Hydrolases | 29 |
| catalytic domain | 23 | (Trans)glycosidases | 27 |
| **S. cerevisiae** |  | **A. thaliana** |  |
| Calcium ATPase | 6 | Protein kinase-like (PK-like) | 123 |
| (Trans)glycosidases | 4 | Cytochrome P450 | 93 |
| Glutaminase/Asparaginase | 4 | P-loop containing nucleoside triphosphate hydrolases | 84 |
| Histone-fold | 4 | RNI-like | 64 |
| Oligoxyloglucan reducing end-specific cellobiohydrolase | 4 | UDP-Glycosyltransferase/glycogen phosphorylase | 53 |
| ATP-binding domain N | 3 | Cysteine-rich domain | 48 |
| Aquaporin-like | 3 | TRAF domain-like | 43 |
| HAD-like | 3 | Bifunctional inhibitor/lipid-transfer protein/seed storage 2S albumin | 40 |
| Metal cation-transporting ATPase | 3 | Plant invertase/pectin methylesterase inhibitor | 38 |
| transduction domain A | 3 | NAD(P)-binding Rossmann-fold domains | 35 |
| transmembrane domain M | 3 | Toll/Interleukin receptor TIR domain | 35 |
| BolA-like | 2 | alpha/beta-Hydrolases | 35 |
| C-terminal domain | 2 | F-box domain | 31 |
| Carbon-nitrogen hydrolase | 2 | Mannose-binding lectins | 31 |
| Chalcone isomerase | 2 | RING/U-box | 29 |
